# C-Terminal Interactors of the AMPA Receptor Auxiliary Subunit Shisa9

**DOI:** 10.1371/journal.pone.0087360

**Published:** 2014-02-03

**Authors:** Anna R. Karataeva, Remco V. Klaassen, Jasper Ströder, Marta Ruiperez-Alonso, Johannes J. J. Hjorth, Pim van Nierop, Sabine Spijker, Huibert D. Mansvelder, August B. Smit

**Affiliations:** 1 Department of Molecular and Cellular Neurobiology, Center for Neurogenomics and Cognitive Research, Neuroscience Campus Amsterdam, Vrije Universiteit University, Amsterdam, The Netherlands; 2 Department of Integrative Neurophysiology, Center for Neurogenomics and Cognitive Research, Neuroscience Campus Amsterdam, Vrije Universiteit University, Amsterdam, The Netherlands; Institute for Interdisciplinary Neuroscience, France

## Abstract

Shisa9 (initially named CKAMP44) has been identified as auxiliary subunit of the AMPA-type glutamate receptors and was shown to modulate its physiological properties. Shisa9 is a type-I transmembrane protein and contains a C-terminal PDZ domain that potentially interacts with cytosolic proteins. In this study, we performed a yeast two-hybrid screening that yielded eight PDZ domain-containing interactors of Shisa9, which were independently validated. The identified interactors are known scaffolding proteins residing in the neuronal postsynaptic density. To test whether C-terminal scaffolding interactions of Shisa9 affect synaptic AMPA receptor function in the hippocampus, we disrupted these interactions using a Shisa9 C-terminal mimetic peptide. In the absence of scaffolding interactions of Shisa9, glutamatergic AMPA receptor-mediated synaptic currents in the lateral perforant path of the mouse hippocampus had a faster decay time, and paired-pulse facilitation was reduced. Furthermore, disruption of the PDZ interactions between Shisa9 and its binding partners affected hippocampal network activity. Taken together, our data identifies novel interaction partners of Shisa9, and shows that the C-terminal interactions of Shisa9 through its PDZ domain interaction motif are important for AMPA receptor synaptic and network functions.

## Introduction

The AMPA-type glutamate receptor (AMPAR) is widely expressed in the brain and mediates the majority of fast excitatory neurotransmission. The AMPAR is a transmembrane glutamate-gated ion channel comprised of 4 pore-forming subunits GluA1–4 [1]. The subunit stoichiometry determines aspects of AMPAR function, including channel conductance, receptor trafficking and subcellular localization [2]. In addition, a group of auxiliary transmembrane proteins regulates membrane expression and function of the AMPAR. These include TARPs [3], and the Cornichons CHIN-2 and CHIN-3 [4]. Recently, the AMPAR-modulating protein Shisa9, initially named CKAMP44, was added to this list [5]. Shisa9 modulates synaptic short-term plasticity by influencing kinetics and channel properties of the AMPAR via direct interaction. Shisa9 is a type-I transmembrane protein that is localized postsynaptically and predominantly expressed in neurons of the hippocampal dentate gyrus and in the cerebral cortex. Shisa9 belongs to the Shisa protein family [6], which is characterized by the presence of a cystine-rich motif in the extracellular domain and a PDZ type II binding motif Glu-Val-Thr-Val (EVTV) at the distal intracellular C-terminus.

AMPARs are known to anchor at their postsynaptic site in order to align transmitter reception with the presynaptic transmitter release. Anchoring of AMPARs at the postsynaptic density (PSD) occurs mostly through proteins that associate with the intracellular domains of AMPAR subunits. For instance, PDZ domain-containing scaffolding proteins interact directly with the AMPAR GluA2 subunit, through binding of GRIP1 [7] and PICK1 [8], and to GluA1 via SAP-97 [9]. Alternatively, AMPARs bind indirectly to the postsynaptic scaffold proteins, e.g., PSD95, via direct interaction with TARP that simultaneously binds to AMPARs and PSD95 [10], [11]. Albeit that AMPARs are anchored postsynaptically, they are highly mobile receptors. They undergo constitutive and activity-dependent translocation to, and removal from, synapses, which is determined by guided lateral diffusion [12], and receptor endo-/exocytosis events [13]. These processes also involve AMPAR associated proteins. For instance, TARP is involved in the lateral insertion of new AMPARs at the postsynaptic membrane [14]. Changing the number of AMPARs residing at the postsynaptic membrane underlies synaptic plasticity and the expression of memory [15]; increases in the amount and function of synaptic AMPAR lead to LTP [16], [17] and, conversely, removal of AMPAR from postsynaptic density mediates LTD [18], [19]. It is conceivable that auxiliary subunits transiently interacting with AMPARs are of importance for anchoring the receptor at the postsynaptic site.

In this study we aimed at identifying cytosolic C-terminal interacting partners of Shisa9, as they might be important for anchoring the protein at the postsynaptic membrane, and elucidating the involvement of these interactors in AMPAR synaptic and network functions.

## Materials and Methods

### Yeast two-hybrid screen

The yeast two-hybrid screen was performed according to methods described by Walhout and Vidal [20]. For bait-construction, the PCR-amplified Shisa9 C-terminal domain (amino acids 172(KLGL)-424(EVTV) of NCBI Refseq NP_082553.2) was inserted into the *Eco*RI–*Sal*I-digested pBD-GAL4 vector (Stratagene). The screen was performed by high-efficiency transformation of a pACT2-plasmid containing mouse brain Matchmaker cDNA library (Clontech) into bait construct-positive PJ69-2a yeast cells (displaying no intrinsic reporter activity). Transformed cells were selected for 4 to 6 days on plates supplemented with 3 mM 3-amino-1,2,3-triazole and lacking amino acids Leu, Trp and His (–LTH), followed by a secondary selection under Leu-, Trp- and Ade-depleted conditions (–LTA). Growth-positive transformants were picked on days 4, 7 and 10, and subjected to another 15 days of –LTA selection.

For the prey protein identification, yeast colonies were resuspended in 15 µL of Zymolyase solution (4 mg/mL Zymolyase T-100 (Seikagaku corporation), 1.2 M Sorbitol and 0.1 M sodium phosphate buffer (pH 7.5)), incubated for 1 hour at 37°C, and heated to 98°C for 10 min. pACT2 inserts were PCR-amplified from the crude-lysate (forward: 5′-GATGATGAAGATACCCCACCAAACCC-3′, reverse: 5′-GCACGATGCACAGTTGAAGTGAACTTG-3′), used as template in BigDye™ terminator 3.1 sequence reactions (Applied Biosystems) (primer: 5′-TCTGTATGGCTTACCCATACGATGTTCC-3′), and analyzed on an Applied Biosystems 3730 DNA Analyzer. Sequence files were blasted against the international protein index database (ipi.MOUSE.v3.37), frame-checked and validated to contain no in-frame stop-codons upstream of the prey-protein coding region.

### Direct two-hybrid assay

The Shisa9-cdΔEVTV bait-construct was PCR amplified, inserted into the *Eco*RI–*Sal*I-digested pBD-GAL4 vector, and transformed into the PJ69-2a yeast strain. Selected prey-clones were rescued from yeast using the RPM kit (MP Biochemicals, used according to the manufacturer's instructions), amplified in *Escherichia coli* (DH5αF), and transformed into the PJ69-2α yeast strain. The identity of each isolated clone was confirmed by sequence analysis, and by blasting against the NCBI reference proteins database.

Bait and prey transformants were grown under respectively -T and -L selective conditions, diluted to an OD_600_ of 0.5, mixed according to the direct two-hybrid matrix, and spotted on rich medium Yeast extract Peptone Dextrose plates. The cells were allowed to grow for 48 hours, followed by replica-stamping onto –LT selective medium. After 3 days the plates were analyzed for cell-growth, replica-stamped onto -LTAH plates (high stringency selection) and incubated for 10 days. Cell-growth was recorded at days 4, 7 and 10. The identities of the bait and prey proteins were re-confirmed at the end of the direct two-hybrid assay by insert amplification and sequence analysis (as described in the yeast two-hybrid screen section).

### DNA constructs

cDNA fragments encoding mouse full length and HA-tagged Shisa9 (WT and ΔEVTV) were amplified by PCR from a previously designed plasmid containing HA-tagged Shisa9. The HA-tag was introduced between the signal peptide and the N-terminus. PCR products were subcloned into the pTRCGW-CMV-IRES2-EGFP-Dest vector. The mouse cDNA encoding full length proteins of putative Shisa9 interactors (PSD93, PSD95, MPP5, PICK1, GRIP1, GIPC1, Lin7b, Dynlt3) were amplified by PCR and subcloned into pcDNA3.2V5/Dest vector (Invitrogen) to obtain V5-tagged proteins. All constructs were sequence verified and used for transfection of HEK293T cells.

### Transfection of HEK293T cells

HEK293T cells were transfected using PEI 2500 (Polysciences). Cells were passed the day before transfection in DMEM media (Gibco), 10% fetal bovine serum (Invitrogen), 1% Penicillin-Streptomycin (Gibco) in 10 cm dishes. On the day of transfection cells were 60–70% confluent. The medium was refreshed 2 h before transfection; 5 µg DNA was mixed with 250 µL PBS, after which 35 µL PEI 2500 was added to the DNA-PBS mixture. The transfection mixture was gently vortexed, incubated for 10 min at RT and drop-wise added to HEK293T cells. After transfection (48 h), cells were harvested in 1 mL of lysis buffer (25 mM HEPES/NaOH, pH 7.4, 150 mM NaCl, EDTA-free protease inhibitor cocktail (Roche)) with 1% n-dodecyl β-d-maltoside (DDM) (Thermo Scientific) or 1% Triton X-100 (Roche, for PICK1), incubated (45 min, rotating) at 4°C, spun down at 20,800 *g* for 10 min at 4°C. The obtained supernatant was used for co-immunoprecipitation.

### Co-immunoprecipitation from HEK293T cells

Anti-HA-tag antibody (2 µg; ab9110, Abcam) was added to the obtained HEK293T cells lysates and incubated (overnight, rotating) at 4°C. Subsequently, protein A/G beads (30 µL; Santa Cruz) were added and samples were incubated (1 h, rotating) at 4°C and washed 3 times with lysis buffer containing 0.1% TritonX-100. SDS sample buffer (50 µL) containing 10% 2-mercaptoethanol was added to the obtained pellets and boiled for 5 min prior to analysis using SDS-polyacrylamide gel electrophoresis and immunoblotting.

### Animals

C57Bl6J mice (Charles River), were housed 7 a.m. lights on/7 p.m. lights off, with water and food ad libitum (for immunoprecipitations: male and female of >10 weeks; for electrophysiology: males of 2–4 weeks) were handled in accordance to the Dutch law using a protocol approved by the Animal Ethics Committee of the VU University Amsterdam.

### Co-immunoprecipitation from mouse hippocampus and cortex

Mouse cortex or hippocampus was homogenized with a potter and piston at 900 rpm on ice, and twelve times up and down motion in 30 mL homogenization buffer (25 mM HEPES/NaOH (pH 7.4), 0.32 M sucrose, 1× Roche protease inhibitor). The extract was centrifuged at 1,000 *g*, 10 min at 4°C. The supernatant was removed, centrifuged at 100,000 *g*, 2 h to obtain a pellet P2-fraction, which was resuspended in HEPES buffer to 10 µg/µL protein, and mixed with an equal volume of lysis buffer with 2% DDM. After incubation (45 min rotation) at 4°C, the sample was centrifuged (20,000 *g* 15 min) at 4°C. The pellet was resuspended in lysis buffer with 1% DDM (300 µL), incubated for another 45 min rotation at 4°C, and again centrifuged. The obtained supernatants (1425 µL, 6 mg protein) were pooled, and anti-Shisa9 antibody (12 µg, PA5-21058, Thermo Scientific) was added and incubated overnight (rotation at 4°C). Agarose-protein A/G beads were added and incubated for 1 h at 4°C. After washing for 4 times in lysis buffer with 0.1% DDM, proteins were eluted off the beads with 60 µL SDS sample buffer, and were loaded (10 µL) on a Criterion Precast gel (BioRad).

### Western blotting

Immunoblotting was done overnight at 40 V onto PVDF membrane (BioRad). For immunostaining of co-immunoprecipitation samples from HEK293T cells the following antibodies were used: anti-V5 (Abcam, 1∶1,000), anti-HA (3F10, Roche, 1∶1,000) in 5% milk TBST, incubation was done overnight at 4°C on a shaking platform. For co-immunoprecipitation samples from brain anti-PSD95 antibody (75-028, Neuromab, 1∶10,000) was used. The secondary antibodies used were goat-anti-mouse-HRP (DAKO, for anti-V5 and anti-PSD95) and goat-anti-rat-AP (Southern Biotech, for anti-HA). The membranes were imaged by means of enhanced chemifluorescence (Amersham) or enhanced chemiluminescence femto (Thermo Scientific) according to the manufacturer's instructions.

### Purification of recombinant PSD95

Mouse His-tagged PSD95 was produced in *E.coli* BL21AI strain (Invitrogen) transformed with pDEST17-PSD95 plasmid. The expression of PSD95 was induced at OD_600_ = 0.6–0.8 with 0.2% arabinose (Sigma). Cells were harvested 3 h after induction by spinning down at 20,000 *g* for 30 min at 4°C. Pellets were resuspended in lysis buffer 25 mM HEPES/NaOH (pH 7.4), 150 mM NaCl with 25 mM Imidazole, frozen in liquid nitrogen and stored at −80°C. Upon use, resuspended pellets were thawed (30°C) and EDTA-free protease inhibitor cocktail (Roche) was added. Cells were cracked by means of the One Shot system (Constant Systems Limited) at 1.7 kbar, repeated 3 times, after which lysates were spun down at 20,000 *g*, 15 min at 4°C. Supernatant was filtered (0.45 µm filter; Millipore) before loading on 1 mL HisTrap column (GE Healthcare) equilibrated with lysis buffer containing 25 mM Imidazole. Purification was performed on AKTA system (GE Healthcare). PSD95 was eluted from the column with a linear gradient of Imidazole up to 500 mM. Fractions were collected, pooled together, frozen in liquid nitrogen and stored at −80°C. PSD95 was concentrated (Amicon ultracentrifuge filter unit 10,000 MW cut-off; Millipore) to 1.4 mg/mL, aliquoted, frozen in liquid nitrogen and stored at −80°C until needed.

### Peptide competition assay

Biotin- and TAT-Shisa9WT peptides identical to last 19 amino acids of the C-terminal part of Shisa9 were used, and 0.5 µM biotinylated Shisa9WT peptide (biotin-HFPPTQPYFITNSKTEVTV) or Shisa9ΔEVTV peptide (biotin-HFPPTQPYFITNSKT; GenScript Corporation) were incubated with NeutrAvidin beads (100 µL; Thermo Scientific) for 10 min at RT while rotating. Unbound peptide was washed away (3 times) with lysis buffer containing 0.05% Tween-20. Recombinant PSD95 (0.1 µM) was added in a total volume of 1 mL and incubated for another 10 min at RT while rotating. The TAT-tagged Shisa9 peptide (10 µM; TAT-Shisa9WT – TAT-HFPPTQPYFITNSKTEVTV; TAT-Shisa9ΔEVTV – TAT-HFPPTQPYFITNSKT or TAT-scrambled – TAT-YPNETKQTIFVSVTPHPFT, GenScript Corporation) was added to the beads-PSD95 mixture and incubation continued for another 2 h at RT. Unbound PSD95 was washed away with cold lysis buffer containing 0.1% Triton X-100 (Roche), washing was performed 4 times, and at the last step beads were transferred to a new tube. To the obtained bead pellet 75 µL SDS-sample buffer was added and boiled for 5 min prior to SDS-PAGE. Samples were loaded on a Criterion Precast gel (BioRad) and the PSD95 protein band was visualized by means of 2,2,2-trichloroethanol present in the precast gels.

### Synaptic plasticity and network oscillations

Acute horizontal hippocampal slices, 300 µm or 400 µm thick, were prepared from either 21 to 30 or 12 to 17 days-old C57BL/6 mice to perform either synaptic plasticity or network oscillations experiments, respectively. After decapitation, the brain was quickly removed and sliced in ice cold artificial cerebro-spinal fluid (aCSF) containing (in mM): 110 choline chloride, 25 NaHCO_3_, 11.6 Na-ascorbate, 10 D-glucose, 7 MgCl_2_, 3.1 Na-pyruvate, 2.5 KCl, 1.25 NaH_2_PO_4_, 0.5 CaCl_2_ for synaptic plasticity recordings and 126 NaCl, 3 KCl, 10 D-glucose, 26 NaHCO_3_, 1.2 NaH_2_PO_4_, 1 CaCl_2_ and 3 MgSO_4_, for oscillations recordings. In both cases, aCSFs were carboxygenated with 95% O_2_ and 5% CO_2_ (pH 7.4). Slices were transferred to a bath of carboxygenated modified aCSF containing (in mM): 2 CaCl_2_, 1 MgCl_2_ and 25 Glucose for the synaptic recordings or 2 CaCl_2_ and 2 MgSO_4_ for the oscillations recordings. Slices were incubated for at least 1 h prior to recording with 10 µM of TAT-Shisa9WT, TAT-Shisa9ΔEVTV or TAT-scrambled peptide. Experiments were performed at 31±1°C. Whole cell recordings of dentate gyrus granule cells were performed using borosilicate electrodes with a resistance of 3–5 MΩ filled with internal solution containing (in mM): 120 Cs-gluconate, 10 CsCl, 8 NaCl, 10 HEPES, 10 phosphocreatine-Na, 0.3 Na_3_GTP, 2 Mg-ATP, 0.2 EGTA, and 4% Biocytin (pH 7.3). Input resistances were monitored throughout recordings. Lateral perforant path inputs were stimulated using electrical stimulation. Local field potentials were measured at the CA3 hippocampal area by means of multi-electrode arrays consisting of 60 electrodes spaced at 100 µm. Oscillations were chemically induced by the addition of 3,5-dihydroxyphenylglycine (DHPG) (10 µM). Data analysis was performed by custom-made software developed in Matlab®.

### Statistics

Data is presented as average ± SEM. Statistical significance was tested with the student's t-test (α = 0.05). Correction for multiple comparisons was applied for the oscillations' analysis. A two-way ANOVA was performed for the paired-pulse-ratio analysis using Bonferroni post-hoc testing. Significance is marked with asterisks as *** *p*<0.0001, ** *p*<0.01 and * *p*<0.05. All data was normally distributed.

## Results

### Shisa9 interacts with PDZ domain-containing proteins in a PDZ-ligand motif-dependent manner

**Figure 1:**
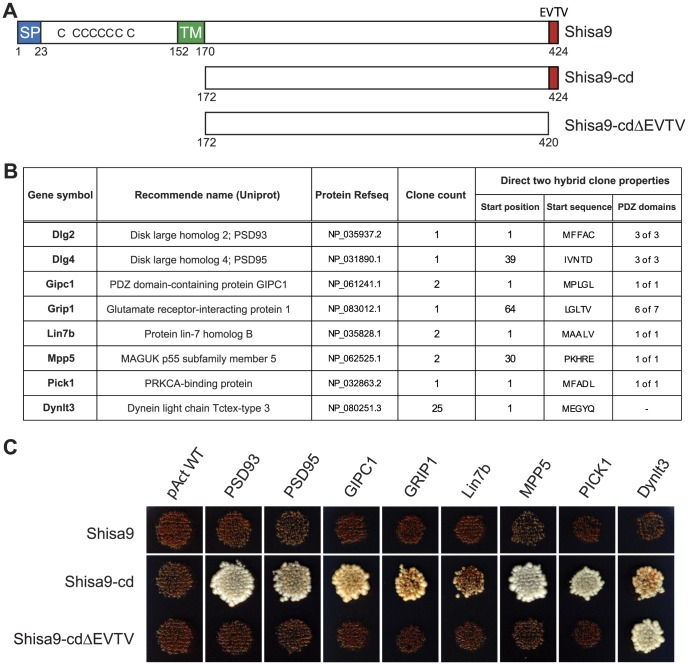
The cytoplasmic side of Shisa9 interacts with multiple PDZ domain-containing proteins in a PDZ-ligand motif dependent manner. **A.** Schematic representation of Shisa9 and the two Shisa9 cytoplasmic domains (cd) used within the yeast two-hybrid screen and direct two-hybrid assay (SP, signal peptide; TM, transmembrane domain; EVTV, C-terminal PDZ-ligand motif. **B.** Putative Shisa9 interactors (Gene symbol, recommended Uniprot name) selected for validation, as identified by yeast two-hybrid. The “clone count” represents the number of hits in the screen, the “start position” refers to the first amino acid of the protein's reference sequence (Protein Refseq) conserved within the direct two-hybrid clone, and the “PDZ domains” column lists the number of complete PDZ domains anticipated within that clone. **C.** Direct two-hybrid assay performed under stringent nutritional selection (–LTAH). The red coloration results from the cell's inability to activate the adenine reporter gene.

To identify cytosolic proteins potentially involved in the interaction with Shisa9, 6.2×10^6^ clones of a mouse brain cDNA library were screened in a yeast two-hybrid system using the Shisa9 cytoplasmic domain (cd) as bait (Fig. 1a). Out of 426 yeast cell transformants that induced cell growth under nutritional selective conditions, 384 were processed for prey-protein identification by prey plasmid isolation and sequencing (see Methods). Blasting prey library-plasmids against the IPI protein-database (ipi.MOUSE.v3.37) resulted in the identification of 146 cDNA clones (E-value<0.001), 84 of which featured both a correct reading frame and a lack of internal stop codons. Combined, the collapsed sequences represented 43 different putative Shisa9-cd interactors (Table S1), including several proteins that contained the anticipated PDZ domains. For follow-up studies, the postsynaptic scaffold-components PSD93, PSD95, MPP5 and GRIP1 were selected, in addition to synaptic-trafficking proteins PICK1, Lin7b and GIPC1 (Fig. 1b). Dynlt3, a well represented, but PDZ domain-lacking protein, was also taken along. The specificity of putative Shisa9 interactors was confirmed with a direct two-hybrid assay using representative clones of each protein and the empty bait vector as control (Fig. 1b–c). None of the interactors was able to induce cell-growth in the absence of the Shisa9-cd while cultured under high-stringent selective conditions (–Leu, –Trp, –His, –Ade).

To establish the involvement of the Shisa9 C-terminal PDZ-ligand motif in protein-protein interaction, we re-tested interaction after deletion of the PDZ interaction motif. Indeed removal of the C-terminal EVTV sequence completely disrupted cell growth for PDZ domain-containing proteins, while in the case of Dynlt3, cell growth did occur (Fig. 1a, c). This confirms that the distal EVTV motif indeed is involved in, and essential for the specific interaction of Shisa9 with all identified PDZ domain-containing binding partners.

### Independent validation of putative Shisa9 interactors by means of co-immunoprecipitation from HEK293T cells

**Figure 2:**
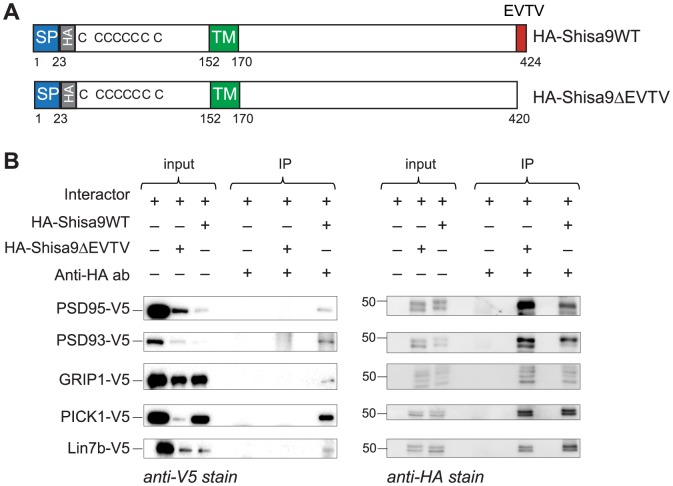
Validation of interaction between Shisa9 and its putative interactors by means of co-immunoprecipitation from HEK293T cells. **A.** Schematic view of Shisa9-constructs used in co-immunoprecipitations. SP, signal sequence; TM, transmembrane domain; HA, HA-tag; EVTV, PDZ-ligand motif. **B.** Co-immunoprecipitation of Shisa9-interactor complexes from HEK293T cells. HA-Shisa9WT and HA-Shisa9ΔEVTV were overexpressed in HEK293T cells in combination with interacting proteins (one at a time). Anti-HA-tag antibody was added to immunoprecipitate HA-Shisa9-interactor complexes. Obtained samples were resolved on SDS-PAGE, western blotted and immunostained with anti-V5 antibody against V5-tagged interactors. Shisa9WT co-immunoprecipitates with PSD95, PSD93, GRIP1, PICK1 and Lin7b proteins, whereas Shisa9ΔEVTV lost the possibility to establish the interaction with named proteins (left panel). The right panel shows the same membranes as in the left panel stained with the anti-HA antibody in order to visualize the presence of Shisa9 in the immunoprecipitated samples. The 50 kDa band is indicated.

To validate the interactions identified in the yeast two-hybrid system, we overexpressed HA-tagged Shisa9WT or HA-Shisa9ΔEVTV proteins and V5-tagged interactors in HEK293T cells and performed co-immunoprecipitations using anti-HA antibody. We confirmed that Shisa9 interacts with PSD95, PSD93, PICK1, GRIP1 and Lin7b via the PDZ domain present in these interacting partners, since Shisa9ΔEVTV lost the possibility to establish an interaction with these proteins. MPP5, Dynlt3 and GIPC1 failed to show interaction with Shisa9 in the co-immunoprecipitations (Fig. 2). Given that the tested interactors of Shisa9 are highly expressed in PSD, we conclude that PSD95, PSD93, PICK1, GRIP1 and Lin7b might represent true interacting partners of Shisa9.

### PSD95 is present in brain-derived Shisa9 complexes

**Figure 3:**
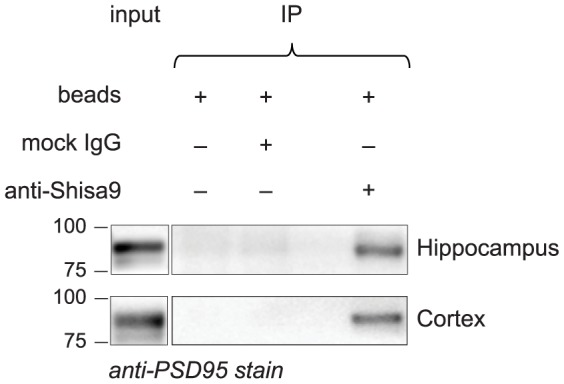
Validation of the interaction of Shisa9-PSD95 the brain. Shisa9 forms a complex with PSD95 in the hippocampus and cortex. Anti-Shisa9 antibody was added to the mouse cortex and hippocampus lysates to immunoprecipitate native Shisa9 complexes. Obtained samples were resolved on SDS-PAGE, western blotted and immunostained with anti-PSD95 antibody. The 75 and 100 kDa bands are indicated.

The PSD is a synaptic protein structure that is very densely packed, which makes it difficult to bring into solution. For this reason, proteins that were identified in this study as Shisa9 interactors have not been identified in our previous experiments based on mass spectrometry analysis [5]. Here we used immunoprecipitation of native Shisa9 complexes from brain tissue followed by immunoblotting, which is a more sensitive technique than mass-spectrometry for the identification of a pool of endogenously interacting proteins. We performed this experiment on two different brain regions, the hippocampus and the cortex. We were able to demonstrate that Shisa9 binds to PSD95 in hippocampus and in cortex (Fig. 3).

### A TAT-tagged peptide of the Shisa9 C-terminus disrupts interaction between Shisa9 and PSD95

To establish whether the interaction of Shisa9 and PSD95 has a role in AMPAR function in hippocampus, and to resolve what these interactions of Shisa9 might mean to synaptic function, we aimed at disrupting the interaction in acute hippocampal brain slices. For this, we generated a synthetic peptide that fuses the TAT-sequence (GRKKRRQRRRPQ) to the 19 C-terminal amino acid residues of Shisa9. This mimetic peptide is designed to compete for the C-terminal interaction with the interactor of Shisa9. The TAT sequence carries fused sequences into neuronal cells *in vivo* [21], [22].

**Figure 4:**
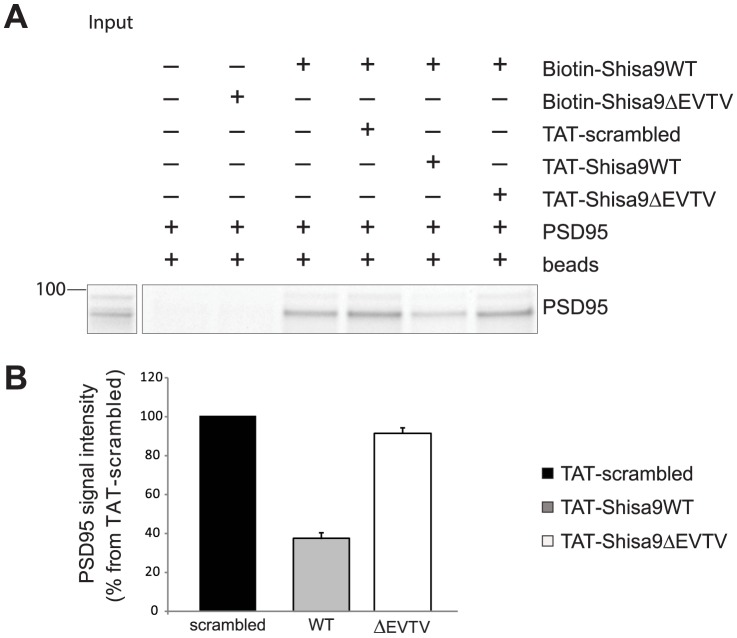
TAT-Shisa9 C-terminus mimetic peptide disrupts interaction between Shisa9 and recombinant PSD95. **A.** The TAT-tagged Shisa9WT C-terminal mimetic peptide, but not the TAT-scrambled and TAT-Shisa9ΔEVTV peptide, competes off the interaction between recombinant PSD95 and the biotinylated Shisa9WT peptide. The 100 kDa band is indicated. **B.** Quantification of the PSD95 band in the presence of TAT-scrambled, TAT-Shisa9WT or TAT-Shisa9ΔEVTV peptide. PSD95 band intensities were normalized to the intensity of PSD95 band in TAT-scrambled peptide lane. All experiments were performed 3 times.

We first tested whether this C-terminal TAT-mimetic peptide of Shisa9 is able to compete for interaction with PSD95. For this, biotinylated Shisa9WT peptide coupled to NeutrAvidin beads, was allowed to interact with recombinant mouse PSD95 protein via interaction of the EVTV motif in the peptide and the PDZ domain in PSD95. Subsequently, a molar excess of TAT-tagged Shisa9WT mimetic peptide was added in order to disrupt the interaction between PSD95 and biotinylated Shisa9 peptide. As control, we used a TAT-tagged Shisa9 peptide lacking the EVTV stretch (analogous to the Shisa9ΔEVTV protein used in Figs. 1–2) and a TAT-scrambled peptide. Samples were subjected to analysis by SDS-PAGE and stained with trichloroethylene (Fig. 4). The intensities of PSD95 were quantified by Image Lab software (BioRad) and normalized to the amount of PSD95 in the TAT-scrambled lane. The TAT-Shisa9WT peptide disrupted approximately 50% of the interaction between the existing PSD95 and the biotinylated Shisa9 peptide, whereas the TAT-Shisa9ΔEVTV peptide was similar to the TAT-scrambled control peptide. This indicates that TAT-Shisa9WT peptide is capable of competing for interaction between PSD95 and Shisa9 (Fig. 4). From this experiment, we extrapolated that a peptide concentration of 10 µM or lower should be used in hippocampal slice experiments to interfere with Shisa9-PSD95 interactions.

### Shisa9-PDZ interactions affect synaptic AMPAR function in hippocampus

**Figure 5:**
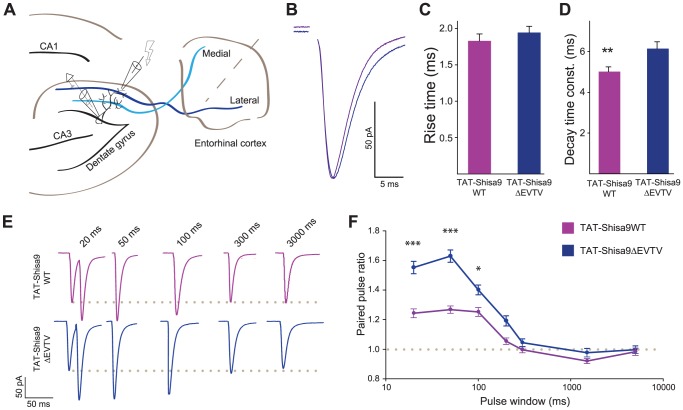
Partial disruption of C-terminals Shisa9 interaction affect AMPAR mediated currents in denate granule cells. **A.** Diagram showing the recording site and the electrically stimulated fibers of the lateral perforant path (dark blue; adapted from [38]. **B.** Example traces of AMPAR-mediated EPSCs after incubation with either the TAT-Shisa9WT (active) or the TAT-Shisa9ΔEVTV (control) peptide. Traces were aligned to the onset of the current. **C, D.** Bar graphs (mean±SEM) summarize the changes in rise- and decay kinetics *vs.* the kinetics of the control pulse. ** *p*<0.01 (Student's t-test). **E.** Representative recordings of a paired-pulse protocol at different stimulation intervals after incubation with either the TAT-Shisa9WT (purple) or the TAT- Shisa9ΔEVTV (blue) peptide. The dotted line indicates the amplitude of the first pulse. Note the decreased paired-pulse facilitation for the TAT-Shisa9WT peptide. **F.** Averages (±SEM) summarizing the differences in paired-pulse ratio facilitation at different inter-event-intervals. *** *p*<0.001, * *p*<0.05 (Post-hoc testing).

In mouse brain, Shisa9 is expressed in the dentate gyrus of the hippocampus [5]. In hippocampal neurons, Shisa9 overexpression prolongs the decay kinetics of AMPAR mediated currents [5]. Given that Shisa9 has PSD interaction partners interacting via PDZ domains, we hypothesized that Shisa9 will exert this function at hippocampal AMPARs when having these protein-protein interactions intact. To test this, we recorded from dentate gyrus granule cells in acute hippocampal slices of the mouse and stimulated glutamatergic projections of the lateral perforant path (LPP; Fig. 5a). We interfered with Shisa9-PDZ interactions by applying the TAT-Shisa9 mimetic peptide (TAT-Shisa9WT), and using the modified peptide (TAT-Shisa9ΔEVTV) as control (see Methods; Fig. 3). Neither the presence of a TAT-scrambled peptide, nor untreated wild type slices showed differences when compared with the TAT-Shisa9ΔEVTV control (Fig. S1). In the presence of the Shisa9-PDZ interfering TAT-Shisa9WT peptide, AMPAR-mediated synaptic currents showed faster deactivation kinetics than in the presence of the control peptide (TAT-Shisa9ΔEVTV (n = 23) 6.15±0.3 ms; TAT-Shisa9WT (n = 21) 5.01±0.2 ms; *p* = 0.007, student's t-test). AMPAR current rise times were not different between the two conditions (TAT-Shisa9ΔEVTV (n = 23) 1.944±0.94 ms; TAT-Shisa9WT (n = 21) 1.829±0.829 ms; *p* = 0.377, student's t-test) (Fig. 5b, c, d). These data show that disrupting C-terminal PDZ domain interactions of Shisa9, through which it interacts with PSD proteins, affects synaptic AMPAR current properties in hippocampus.

AMPAR-mediated glutamatergic synaptic currents in Shisa9 knockout animals show slower recovery from desensitization observed by reduced paired-pulse facilitation in dentate gyrus granule cells [5]. To test whether Shisa9-PDZ interactions are involved in recovery from desensitization of synaptic AMPARs, we tested the effect of the TAT-Shisa9 mimetic peptide on paired-pulse facilitation in whole cell recordings from dentate gyrus granule cells stimulated in the lateral PP. Interference with Shisa9-PDZ interactions reduced paired-pulse facilitation (Fig. 5e, f; Two-way ANOVA: peptide treatment, F(1, 326) = 36.00, p<0.0001; stimulation interval, F (9, 326) = 54.41, p<0.0001; interaction, F (9, 326) = 4.27, p<0.0001; TAT-Shisa9ΔEVTV n = 19, TAT-Shisa9WT n = 23). At the 20, 50 and 100 ms inter-pulse interval, paired-pulse facilitation was significantly (all *p*<0.001) reduced by the TAT-Shisa9WT peptide (50 ms: 1.62±0.04 to 1.27±0.02 in the presence of the control peptide). Thus, these data show that interference with Shisa9-PDZ interactions slowed recovery from desensitization. Together, our findings demonstrate that protein interactions at the C-terminus of Shisa9 affect AMPAR kinetics and synaptic facilitation.

### Shisa9-PDZ interactions shape hippocampal network oscillations

**Figure 6:**
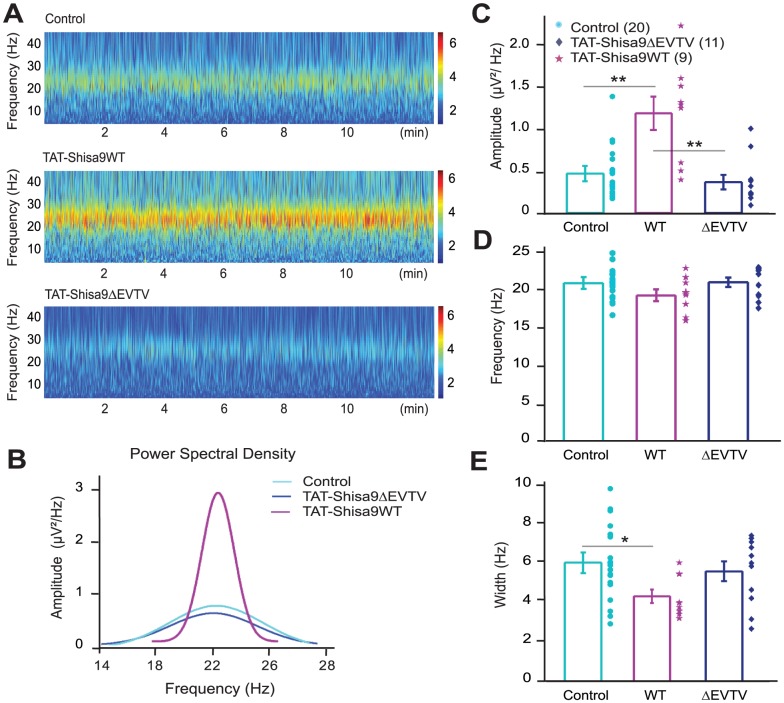
Shisa9 increases the synchrony of DHPG-induced hippocampal oscillations via PDZ domain interactions. **A.** Wavelet display of recorded field potentials of DHPG-induced oscillations under the 3 experimental conditions: Control (no peptide application, top trace), PDZ interacting peptide TAT-Shisa9WT (middle) and inactive form of the peptide TAT-Shisa9ΔEVTV (bottom). Warmer colors indicate higher oscillation amplitude (dimension-less units). **B.** Comparison of the power spectral density of the DHPG-induced oscillations in the 3 experimental conditions: control (light blue), TAT-Shisa9WT (purple), TAT-Shisa9ΔEVTV (dark blue). **C.** TAT-Shisa9WT peptide significantly increases the spectral amplitude of DHPG-induced hippocampal oscillations with respect to no peptide application, as well as with respect to the inactive peptide. **D.** Application of TAT-Shisa9WT peptide has no significant effect on the frequency. **E.** TAT-Shisa9WT peptide significantly narrows the spectral half-width with respect to control conditions. *p<0.05 (Student's t-test).

Synchronization of hippocampal neuronal activity relies on fast synaptic transmission via AMPARs [23], [24]. Given that Shisa9 interactions affect synaptic AMPAR function in hippocampus, we hypothesized that tuning of AMPAR function by Shisa9-PDZ interactions would affect synchronization of neuronal activity. To test this, we recorded network oscillations induced by the metabotropic glutamate receptor agonist DHPG (10 µM) in acute hippocampal slices (Fig. 6a). Interference with Shisa9-PDZ protein interactions by application of the TAT-Shisa9WT peptide altered several parameters of hippocampal network oscillations. The mimetic peptide significantly increased the power spectral amplitude of DHPG-induced hippocampal oscillations both compared to no peptide application (control 0.49±0.07 µV^2^/Hz, n = 20; TAT-Shisa9WT 1.24±0.2 µV^2^/Hz, n = 9, *p* = 0.006, Fig. 6c), as well as compared with inactive peptide (TAT-Shisa9ΔEVTV 0.38±0.09 µV^2^/Hz, n = 11, *p* = 0.0007). Interference with Shisa9-PDZ interactions by the TAT-Shisa9WT peptide showed no significant effect on the average frequency of oscillations (control 21.3±0.4 Hz vs. TAT-Shisa9WT 19.7±0.8 Hz, *p* = 0.07, control vs. TAT-Shisa9ΔEVTV 21.5±0.6 Hz, p = 0.87, Fig. 6d). Application of the mimetic peptide significantly narrowed the spectral half-width with respect to control conditions (control 6.0±0.4 Hz, TAT-Shisa9WT 4.3±0.3 Hz, *p* = 0.01, Fig. 6e). There was no effect on the spectral half-width of the inactive peptide when compared to control conditions (5.5±0.5 Hz for TAT-Shisa9ΔEVTV, *p* = 0.87, Fig. 6e). These data show that PDZ protein interactions of Shisa9 that tune synaptic AMPAR function are involved in setting the properties of hippocampal neuronal network activity and synchronization.

## Discussion

The aim of this study was to identify novel cytosolic Shisa9-interacting proteins, and to establish the relevance of these protein-protein interactions for Shisa9-mediated modulation of glutamatergic synaptic transmission. We have previously reported that Shisa9 is enriched within the postsynaptic density, a protein-packed structure that is notoriously difficult to solubilize while maintaining protein complex integrity, a requirement for immunoprecipitation-based proteomics. The yeast two-hybrid approach to interactor identification circumvents these limitations, while offering improved chance at discovering more transient associations.

In this study, we successfully applied the yeast two-hybrid method in the identification of novel putative Shisa9 interacting proteins. Our findings confirm that Shisa9 can associate with several PDZ domain-containing proteins, such as PSD95, and that this binding is dependent upon Shisa9's distal PDZ-ligand motif (EVTV). The well-established importance of PDZ domain-containing proteins in glutamatergic synaptic plasticity, ranging from receptor trafficking to receptor immobilization/scaffolding [25], and the clearly defined site of Shisa9 association, led us to focus in the follow-up characterization on these interactors.

In the two-hybrid screening using the Shisa9 intracellular domain, we identified 43 putative binding partners. We selected proteins based on the presence of a PDZ domain (PSD95, PSD93, MPP5, PICK1, GRIP1, Lin7b and GIPC1). These proteins are all well known for their presence in the postsynaptic density [26]–[29]. This indicated that Shisa9 could potentially interact with PSD scaffold proteins. In addition, we selected Dynlt3, which does not have a PDZ domain. All selected proteins were tested for autoactivation in a direct mating assay, and were confirmed to be Shisa9 interactors (Fig. 1). Furthermore, we created the Shisa9ΔEVTV mutant, which lacks the PDZ-binding motif. We showed that this mutant loses interaction with PDZ domain-containing proteins, but not with Dynlt3. This way, we corroborated that interaction between Shisa9 and its partners occurs via EVTV-PDZ domain interaction and that interaction with Dynlt3 is via a different interaction site. To independently verify these putative interactors, we confirmed by co-immunoprecipitation in HEK293T cells that Shisa9 interacts with PSD95, PSD93, PICK1, GRIP1 and Lin7b specifically via its PDZ domain, since Shisa9ΔEVTV completely lost interaction with these proteins (Fig. 2).

We identified PSD95 in Shisa9 complexes derived from hippocampus and cortex (Fig. 3). We visualized PSD95 by means of immunoblotting of immunoprecipitation-samples. Immunoblotting is a more sensitive method of protein identification than mass spectrometry and probably explains why PSD95 was not previously found [5]. The fact that the other Shisa9 protein interactors were not found using this method does not exclude them from being binding partners in the PSD *in vivo*, but rather suggests that these could be regulated in a plasticity-dependent manner.

We addressed the issue of whether protein interactions through the C-terminus of Shisa9 affect synaptic AMPA receptor function. To resolve this issue, we made use of TAT-fusion peptides, which have been shown to successfully disrupt protein interactions at AMPAR [15], [22], [30]. We interfered with the interaction between Shisa9 and its partners by applying a C-terminal TAT-tagged mimetic Shisa9WT or a control TAT-Shisa9ΔEVTV peptide. We found that C-terminal protein interactions of Shisa9 tune the functional properties of AMPARs. Interfering with the PDZ-interaction between Shisa9 and its binding partners affected basic functional properties of the AMPA receptors: it sped-up de-activation and slowed-down recovery from desensitization (Fig. 5). Our data are in agreement with previous findings, in which paired-pulse ratios and current decay times of the AMPA receptor in hippocampal CA1 neurons were affected by the overexpression of Shisa9 in this area [5]. Knocking out Shisa9 in dentate gyrus granule cells resulted in increased paired-pulse facilitation of the lateral perforant path inputs [5]. We found that only interfering with C-terminal protein interactions of Shisa9 in granule cells, leaving Shisa9 itself unaltered, reduced paired-pulse facilitation. Preventing Shisa9 to engage in C-terminal PDZ interactions apparently alters short-term facilitation in an opposite direction from removing Shisa9 entirely.

Based on our data that Shisa9 and PSD95 interact, these findings suggest that Shisa9 might be involved in anchoring of the AMPA receptors to the PSD. In our experiments, the introduction of the TAT-Shisa9WT peptide may impair the anchoring of the AMPA receptor at the PSD and therefore might affect diffusion of the AMPA receptors in and around the active zone. Our mimetic peptide approach only allows us to suggest that the Shisa9 protein interaction with the scaffold is of importance to AMPAR function and synaptic plasticity (decreased facilitation). The disrupted interaction includes that of Shisa9 with PSD95, but may include other identified PDZ-containing scaffolding proteins, the latter of which cannot be identified easily by immunoprecipitation from brain samples due to the resistance of the PSD to solubilize.

We found that the tuning of functional properties of synaptic AMPAR by Shisa9 and its protein interactions shaped hippocampal neuronal network oscillations. Hippocampal network oscillations are the result of balanced excitatory and inhibitory synaptic transmission [24]. Interference with Shisa9-PDZ interactions increased the power of network oscillations and narrowed the frequency range of oscillations. Possibly, the longer synaptic AMPAR currents with slower decay kinetics that occur when Shisa9-PDZ interactions are intact, allows the hippocampal network to synchronize at a broader range of frequencies, resulting in a wider power spectral density distribution covering more frequencies. Disrupting Shisa9-PDZ interactions would speed up synaptic AMPAR currents and limit the frequency range at which the network synchronizes, and as a result, increases the power at this limited frequency range. Excitatory glutamatergic synaptic inputs received by interneurons, in particular to those that are parvalbumin-positive and cholecystokinin-positive, are important for hippocampal network oscillations [31], [32]. Whether Shisa9 is also expressed by hippocampal interneurons and whether AMPAR kinetics in interneurons is affected by Shisa9 remains to be determined. Regardless, Shisa9 is expressed in dentate gyrus granule cells [5] and we show that synaptic AMPAR current properties in dentate gyrus granule cells are tuned by Shisa9-PDZ protein interactions. Disruption of these Shisa9-PDZ interactions in dentate gyrus neurons may underlie the effects we observed on hippocampal network activity.

The first auxiliary subunit of the AMPA receptor – stargazin (γ2) – was discovered in the late 1990-s [33]. Since then it was shown that stargazin belongs to the family of the transmembrane AMPA receptor regulatory proteins – TARPs [34]. Identification of the TARPs stimulated the discovery of the cohort of AMPAR's auxiliary subunits – CHIN2 and 3 [4], Shisa9 (CKAMP44; [5], SynDIG1 [35], GSG1L [36]. This list of potential AMPA receptor auxiliary subunits has kept growing [36], [37]. The expanding set of auxiliary subunits raises the question how a large number of structurally unrelated and functionally different proteins regulate the AMPA receptors. In this study, we found that the AMPAR interacting protein Shisa9 binds to well-known PSD proteins, and we established the Shisa9-PSD95 interactions to be present in the brain. In addition we found that affecting the anchoring of Shisa9 via its C-terminal tail in brain slices affects AMPAR function, synaptic plasticity and neuronal network synchronization in the hippocampus. This indicates that Shisa9 not only modulates the biophysical properties of the receptor by direct association but also affects function through controlling its synaptic localization.

## Supporting Information

Figure S1 **A. Data show the paired pulse facilitation (mean ± SEM) at different inter-event intervals upon lateral perforant path stimulation (TAT-Shisa9ΔEVTV (n = 9), TAT-scrambled peptide (n = 12), TAT-Shisa9WT (n = 10).** No significant changes could be observed between the tested groups. **B,C.** Bar graphs (mean ± SEM) summarize the data on rise- and decay kinetics between all tested groups (TAT-Shisa9ΔEVTV (n = 9), TAT-scrambled (n = 12), TAT-Shisa9WT (n = 10). The tested groups showed no significant differences.
(EPS)

Table S1 **Putative Shisa9-cd interactors identified by the yeast two-hybrid screening.**
(EPS)
