# Correction: C-Terminal Interactors of the AMPA Receptor Auxiliary Subunit Shisa9

**DOI:** 10.1371/journal.pone.0099280

**Published:** 2014-05-27

**Authors:** The PLOS ONE Staff

Figure 1 is incorrect. In panel C, Shisa9 should be pBD-WT. The authors have provided a corrected version, which can be viewed here.

**Figure 1:**
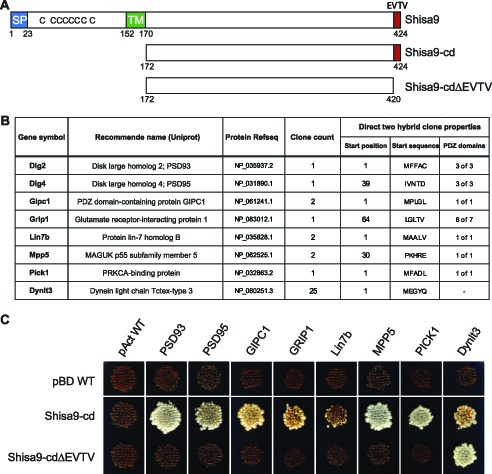
The cytoplasmic side of Shisa9 interacts with multiple PDZ domain-containing proteins in a PDZ-ligand motif dependent manner. **A.** Schematic representation of Shisa9 and the two Shisa9 cytoplasmic domains (cd) used within the yeast two-hybrid screen and direct two-hybrid assay (SP, signal peptide; TM, transmembrane domain; EVTV, C-terminal PDZ-ligand motif. **B.** Putative Shisa9 interactors (Gene symbol, recommended Uniprot name) selected for validation, as identified by yeast two-hybrid. The “clone count” represents the number of hits in the screen, the “start position” refers to the first amino acid of the protein's reference sequence (Protein Refseq) conserved within the direct two-hybrid clone, and the “PDZ domains” column lists the number of complete PDZ domains anticipated within that clone. **C.** Direct two-hybrid assay performed under stringent nutritional selection (–LTAH). The red coloration results from the cell's inability to activate the adenine reporter gene.
